# Effects of empathy on the bidirectional relationships between problematic smartphone use and aggression among secondary school students: a moderated network approach

**DOI:** 10.3389/fpsyt.2024.1359932

**Published:** 2024-02-29

**Authors:** Wenxia Wu, Xinyuan Zou, Qihui Tang, Yanqiang Tao, Shujian Wang, Zijuan Ma, Min Li, Gang Liu

**Affiliations:** ^1^ School of Marxism, Xiamen University, Xiamen, China; ^2^ Faculty of Psychology, Beijing Normal University, Beijing, China; ^3^ School of Psychology, South China Normal University, Guangzhou, China; ^4^ Department of Psychiatry, Affiliated Nanjing Brain Hospital, Nanjing Medical University, Nanjing, China

**Keywords:** problematic smartphone use, aggression, empathy, moderate network analysis, secondary school students

## Abstract

**Background:**

Existing literature on the relationship between problematic smartphone use (PSU) and aggression has primarily focused on examining their unidirectional association, with limited attention paid to the bidirectional nature of this relationship, particularly when considering the role of empathy. This study employs a novel moderated network approach to examine the bidirectional relationship between problematic smartphone use and aggression, while also investigating the moderating mechanism of empathy.

**Methods:**

A total of 2,469 students (49.1% female, *Mean*_age_ = 13.83, *SD*_age_ = 1.48) from 35 junior and senior high schools in Harbin, China, participated in this study. Empathy level, aggressiveness, and PSU symptoms were assessed using the Basic Empathy Scale, the Buss-Warren Aggression Questionnaire, and the Mobile Phone Addiction Index.

**Results:**

Analysis revealed that the relationship between PSU and aggression was complex and bidirectional. The strongest association was observed between “hostility” and “withdrawal/escape”. In addition, “anger” had the highest Expected Influence (EI) in both affective and cognitive moderate network models. An important discovery was also made regarding the conditional effect of “productive loss” and “physical aggression” across different levels of affective empathy. Specifically, at lower levels of affective empathy, a positive bidirectional relationship was found between “productive loss” and “physical aggression”. However, this relationship turned negative and bidirectional at higher levels of affective empathy.

**Conclusion:**

The findings contribute to a more comprehensive understanding of the complex dynamics between PSU and aggression and highlight the need for targeted interventions that promote affective empathy to mitigate the negative consequences of excessive smartphone use.

## 1 Introduction

Problematic smartphone use (PSU), defined as the inability to control mobile phone use, can impair psychological or physiological functioning (1). The mechanism of PSU has been widely explored. Studies have shown that PSU is associated with changes in the structure and function of neurons in the brain and the enhancement of neural reward circuitry (2), which may lead to increased craving and high dependence on mobile phones (3). As adolescents are in a critical developmental stage, they may be particularly vulnerable to PSU. Moreover, the prevalence rate of PSU among adolescents has exceeded 17.7% (4). In particular, PSU can seriously impair the healthy development of adolescents and threaten their mental health (5), social relationships (6), and academic performance (7), highlighting the importance of effective interventions for PSU among adolescents.

Specifically, individuals with PSU tend to show a range of externalizing problems, including aggressive behavior (8). According to Buss and Perry (9), aggressive behavior is identified as all forms of physical or verbal behavior intended to cause harm and includes four sub-dimensions: physical aggression, verbal aggression, hostility, and anger. Noticeably, adolescents can be at high risk of exhibiting aggression (10), with their prevalence rate of aggression reaching 25.0% (11). Alarmingly, aggression in adolescents and children may play an important role in oppositional defiant disorder, conduct disorder, and mood disorders (e.g., depression), which could greatly affect the parent-child relationship and disrupt the normal development of adolescents (12). Furthermore, if left unresolved, persistent aggression is highly associated with low socioeconomic status, unemployment, social isolation, and even criminal behavior later in life (12). Therefore, studies to explore and intervene in adolescent aggression are also warranted.

### 1.1 The bidirectional relationship between aggression and PSU

Considering that PSU shares a similar pattern of behavior with impulse control disorder (13) and that aggression is also closely related to the inability to control impulses (14), it is understandable that a close relationship has been found between PSU and aggression (Karaoglan Yilmaz et al., 20-22).

Specifically, according to the model of self-control, emotion regulation, and aggression, people with higher levels of aggression show lower levels of self-control (15) and have more difficulty controlling their emotional outbursts and hostility (16) when provoked. Indeed, according to the compensation theory of media use, increased hostility and negative emotions may increase the potential to engage in more undesirable mobile use behaviors (e.g., inappropriate talk on the Internet) (17). In fact, higher levels of aggression may be a risk factor and a notable predictor of PSU (13). Consistent with the compensation theory, several studies have demonstrated that aggression can positively predict PSU and the reduction of aggression can contribute to the alleviation of PSU (8), which further provides a basis for support.

On the other hand, PSU is also strongly associated with lower self-control (14). As stated in a previous study, lower self-controllers tend to use mobile phones as a passive coping strategy to deal with their negative emotions, which may lead to the impairment of interpersonal relationships and increase negative emotions such as loneliness, discomfort, and frustration in the long run (18). According to the frustration-aggression theory, people may show more aggressive tendencies and behaviors without adaptive methods to cope with negative feelings and increasing unmet psychological needs (19). Consistent with the frustration-aggression theory, Sahu et al. (20) suggested that higher levels of PSU are associated with a greater risk of behavioral problems (e.g., school bullying). Meanwhile, PSU may reduce the ability to control anger in secondary school students (21), which may also increase aggressive behavior.

To conclude, the relationship between PSU and aggression may be bidirectional. However, the current findings view PSU and aggression simply as the behavioral outcome of a lack of self-control, which may oversimplify the mechanisms underlying these behaviors and hinder our understanding of them. Moreover, in a major contradiction to previous findings (22), recent studies suggest that people with high levels of self-control may also exhibit aggressive behaviors. Indeed, the relationship between aggression and PSU in adolescents and its relationship with other important elements, such as empathy, warrants further exploration.

### 1.2 The role of empathy in the bidirectional relationship between PSU and aggression

Meanwhile, researchers have also found that empathy is an important factor influencing both PSU and aggressive behavior in adolescents (23, 24). Empathy is defined as the ability to perceive and understand the emotions of another individual (25), which includes two sub-dimensions: (1) affective empathy and (2) cognitive empathy. Affective empathy refers to emotional response and empathic behavior toward others (such as concern and warmth towards others), and cognitive empathy refers to awareness of another person’s feelings or thoughts (25). Previous studies have found that low levels of empathy are closely associated with increased aggressive behaviors (26) and a higher risk of PSU (27). Specifically, individuals with low levels of empathy tend to hold less appropriate normative beliefs about aggression and exhibit more aggressive behaviors (26). Meanwhile, deficits in empathy may reduce people’s ability to recognize or respond to others face-to-face, leaving them unable to achieve a sense of security without the mobile phone and increasing their vulnerability to excessive smartphone use (27).

Notably, the relationship between empathy and aggression is still debated. Vachon and Lynam (28) found that cognitive empathy is more strongly correlated with aggression than affective empathy, and van Langen et al. (29) also suggested that high characteristics of aggression are associated with low cognitive empathy. However, another study suggests that affective empathy may have a stronger relationship with physical aggression (30). Apart from this, studies examining the relationship between the two dimensions of empathy and PSU are limited. In addition, research analyzing the relationship between PSU, aggression, and empathy at a symptom level is even more scarce, hindering a comprehensive understanding of these variables and their relationship. Indeed, further research is warranted to clarify the relationship between two dimensions of empathy, PSU, and aggression.

### 1.3 The current study

The present study aims to explore the relationship between empathy, PSU, and aggression using the moderated network approach. Compared to the traditional moderated mediation model approach (31), the network analysis approach can better enclose the dynamic association between symptoms (32). Furthermore, according to Tao et al. (33), the moderated network is an excellent approach to efficiently explore moderating variables. Therefore, we use the Moderated Network Model (MNM) to investigate whether empathy (both affective and cognitive empathy) can moderate the bidirectional relationship between aggression and PSU.

To our knowledge, no studies have compared PSU, aggression, and empathy in secondary school students, especially using a moderated network approach, which motivated us to conduct the current study. Thus, this study tested three hypotheses under two research aims.

Aim 1: To investigate whether there is a bidirectional relationship between PSU and aggression in secondary school students.

Hypothesis 1. As mentioned above, PSU may impair an individual’s ability to control anger (21), which may increase aggressive behavior, and aggression may also positively predict the level of PSU (13). Therefore, we hypothesize that there is a bidirectional relationship between PSU and aggression in secondary school students.

Aim 2: To examine whether empathy (both affective and cognitive empathy) can effectively moderate the relationship between mobile phone addiction and aggression symptoms.

Hypothesis 2. Despite the inconsistency, since previous findings have identified a strong relationship between aggression and both dimensions of empathy (29, 30), we hypothesize that both affective empathy and cognitive empathy can effectively moderate the level of PSU and aggression symptoms.

## 2 Method

### 2.1 Participants

The current study was conducted in Harbin in February 2022, using convenient sampling to collect data. We used an online questionnaire program, Wenjuanxing (https://www.wjx.cn), and collected datasets of 2,469 students (49.1% females, *Mean*_age_ = 13.83, *SD*_age_ =1.48) from 35 junior and senior high schools. Students and their parents have provided signed informed consent before participating in the assessment. This research was reviewed and approved by the Ethics Committee of *** University (reference number: 202112220085).

### 2.2 Measures

First, participants were asked to complete a brief sociodemographic questionnaire. Empathy level, aggressiveness, and PSU symptoms were measured by Basic Empathy Scale (BES), Buss-Warren Aggression Questionnaire (BWAQ), and Mobile Phone Addiction Index (MPAI), respectively.

#### 2.2.1 Basic empathy scale

The original version of the basic empathy scale (BES) compiled by Jolliffe and Farrington (34) consists of 20 items and contains two dimensions: cognitive empathy (e.g., “I can usually detect when someone is depressed”) and affective empathy (e.g., “I often get involved in the emotions of my friends”). The Chinese version was revised by Geng et al. (35), and the two factors remained the same, while four items were deleted. Participants need to answer 16 Likert-style questions scored from 1 (“strongly disagree”) to 5 (“strongly agree”), with a higher score indicating a higher empathy ability. In the current study, BES showed good internal consistency, with a Cronbach’s *α* value of 0.91.

#### 2.2.2 Buss-Warren aggression questionnaire

Buss-Warren aggression questionnaire (BWAQ) (36) consists of 34 Likert-style items scoring from 1 to 5 and contains five dimensions: physical aggression (e.g., “I may hit someone if he or she provokes me”), anger (e.g., “At times I get very angry for no good reason”), verbal aggression (e.g., “I can’t help getting into arguments when people disagree with me”), indirect aggression (e.g., “When someone really irritates me, I might give him or her silent treatment”), and hostility (e.g., “I do not trust strangers who are too friendly”). The Chinese version was revised by Maxwell and Differences (37) and showed good reliability and validity. In the current study, BES showed good internal consistency, with a Cronbach’s *α* value of 0.96.

#### 2.2.3 Mobile phone addiction index

PSU was assessed by the mobile phone addiction index (MPAI). MPAI was compiled by Leung (38). It is a self-report questionnaire composed of 17 5-point Likert-style items. There are four factorial components in MPAI: inability to control craving (e.g., “You find yourself using your phone for longer than you had intended”), feeling anxious and lost (e.g., “If you do not check your messages for a while or your phone is not turned on, you become anxious”), withdrawal or escape (e.g., “When you feel lonely, you use your mobile phone to chat with others”), and productivity loss (e.g., “The time you spent on your phone decreases your work efficiency”). The Chinese version was revised by X. W. Li et al. (39) and showed good validity and reliability. In the current study, the Cronbach’s *α* value of the MPAI was 0.95.

### 2.3 Statistical analysis

R version 4.2.2 (40) performed all statistical analyses. First, we used the least absolute shrinkage and selection operator (LASSO) method for variable screening to reduce false-positive results, sparse the final obtained network structures, and enhance the interpretability of the model (41, 42). Second, the target variables were predicted using the variables and interaction terms screened via the nodewise regression method (43, 44) to estimate the final moderated network model. Two commonly used indexes to evaluate symptom centrality are strength centrality and expected influence (*EI*) (45). Strength centrality is the sum of the absolute values of all edge weights linked with the node, whereas *EI* is simply the sum of those raw edge weights. Since all pairwise edges were positive in this study, there is no difference between using strength and *EI*. The higher centrality indicates this node (symptom) may play a more important role in the network structure. Third, the case-dropping procedure was conducted to test the stability of all edge weights and node *EIs* (46). Case-dropping test can provide the correlation stability coefficient (*CS-C*), which represents the most proportion of samples that could be removed, with a 95% probability that the correlation between the new and the original parameters could be at least 0.70. Generally, the *CS-C* should be ≥ 0.25, preferably ≥ 0.5. More detailed statistical method information and the R packages we used were provided in the **Supplementary Material**.

## 3 Results

### 3.1 Descriptive statistics

Means, standard deviations, skewness, and kurtosis of all dimensions are shown in **Table 1**.

**Table 1:** Descriptive statistic information for all variables.

Construct	Label	*M*	*SD*	Skewness	Kurtosis
Cognitive empathy	BES_C	3.19	0.86	0.26	-1.15
Affective empathy	BES_A	2.73	0.65	-0.17	1.07
Inability to control craving	MPAI1	1.89	0.89	1.13	0.82
Feeling anxious and lost	MPAI2	1.76	0.92	1.44	1.68
Withdrawal/escape	MPAI3	1.97	1.10	1.20	0.72
Productivity loss	MPAI4	1.80	1.02	1.35	1.23
Physical aggression	BWAQ1	1.75	0.89	1.44	1.81
Anger	BWAQ2	2.08	0.94	0.91	0.39
Verbal aggression	BWAQ3	2.29	0.94	0.53	-0.05
Indirect aggression	BWAQ4	1.85	0.88	1.16	1.15
Hostility	BWAQ5	2.07	0.97	0.91	0.29

### 3.2 Moderated network models

**Figure 1:**
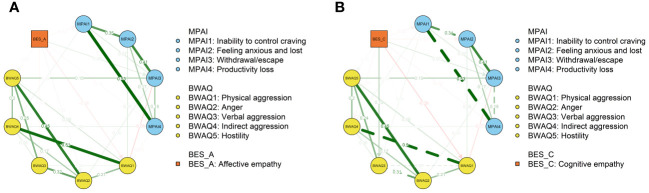
Two MNMs were plotted using the “AND” rule. Green and red lines indicate a positive and negative relationship, respectively, between the two variables. Dashed lines indicate that the relationship between two variables is significantly moderated by the moderating variable, while the solid line indicates that the moderating effect is not moderated (AND rule). The numbers on the lines represent the values of the aggregated nodewise regression. The square represents the moderating variable, and the arrows directly indicate its links with the other variables. **(A)** The network model moderated by affective empathy. **(B)** The network model moderated by cognitive empathy.

The results of variable selection for affective and cognitive empathy based on hierarchical LASSO are presented in **Supplementary Tables S1**, **S2**, respectively. Regression coefficients and 95% confidence intervals for each predictor are shown in **Supplementary Figures S1**, **S2**. The nodewise adjacency matrix and the interaction term matrix of the affective empathy moderated network are shown in **Supplementary Tables S3**, **S4**, while the two matrices of the cognitive empathy moderated network are shown in **Supplementary Tables S5**, **S6**. Two MNMs based on the nodewise and interaction term matrices were visualized in **Figure 1**.

For the affective empathy moderated network, as shown in **Supplementary Table S3**; **Figure 1**, the edges of “inability to control carving” - “productivity loss” (MPAI1-MPAI4) showed the strongest association, followed by “physical aggression” - “indirect aggression” (BWAQ1-BWAQ4), and “hostility” - “anger” (BWAQ5-BWAQ2). Meanwhile, “anger” showed the highest *EI* (top three), followed by “hostility” and “indirect aggression” (see **Supplementary Figure S3**, part A). Between PSU and aggression, the edge is strongest between “hostility” and “withdrawal/escape” (MPAI3-BWAQ5), followed by “physical aggression” and “withdrawal/escape” (BWAQ1-MPAI3), and “physical aggression” and “feeling anxious and lost” (BWAQ1-MPAI2).

For the cognitive empathy moderated network, as shown in **Supplementary Table S5**; **Figure 1**, the edges of “inability to control carving” - “productivity loss” (MPAI1-MPAI4) showed the strongest association, followed by “physical aggression” - “indirect aggression” (BWAQ1-BWAQ4), and “hostility” - “anger” (BWAQ5-BWAQ2). Meanwhile, “anger” showed the highest *EI* (top three), followed by “hostility” and “inability to control carving” (see **Supplementary Figure S3**, part B). Between PSU and aggression, the edge is strongest between “withdrawal/escape” and “hostility” (MPAI3-BWAQ5), followed by “withdrawl/escape” and “physical aggression” and “feeling anxious and lost” and “physical aggression” (MPAI2-BWAQ1).

**Figure 2:**
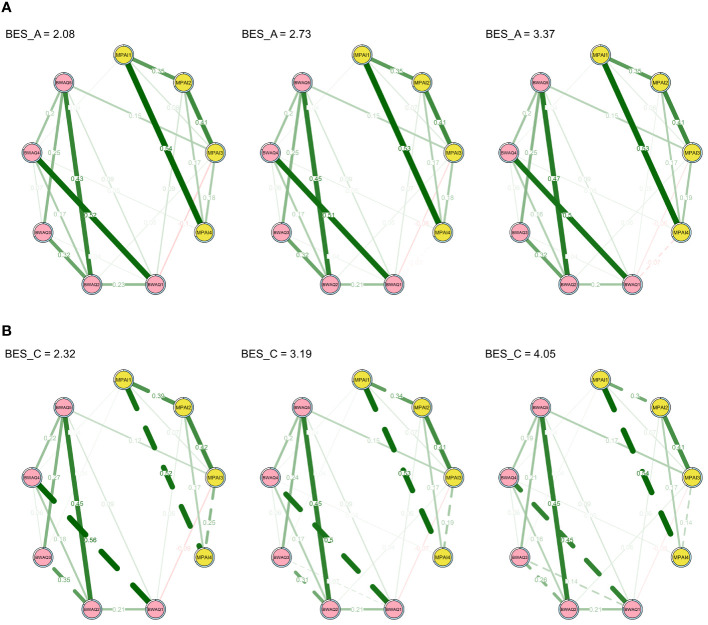
Conditional network models at the values of the mean ± SD of empathy. Plotted with the AND rule and a significance threshold of *p* < .05. **(A)** The affective empathy moderated network model. **(B)** The cognitive empathy moderated network model.

Analyses of the interaction terms showed that the relationship between “productive loss” and “physical aggression” (MPAI4-BWAQ1) was moderated by affective empathy (see **Supplementary Table S4**). In particular, **Figure 2** shows more clearly how network structures change with increasing levels of affective and cognitive empathy. We can see that when the level of affective empathy changed from the mean to one standard deviation above the mean, the correlation between “productivity loss” and “physical aggression” also decreased (part A of **Figure 2**). However, cognitive empathy had no moderating effect on the relationship between PSU and aggression (see **Supplementary Table S6**). Thus, as cognitive empathy increased, there was little change in the association between aggression and PSU symptoms (part B of **Figure 2**).

**Figure 3:**
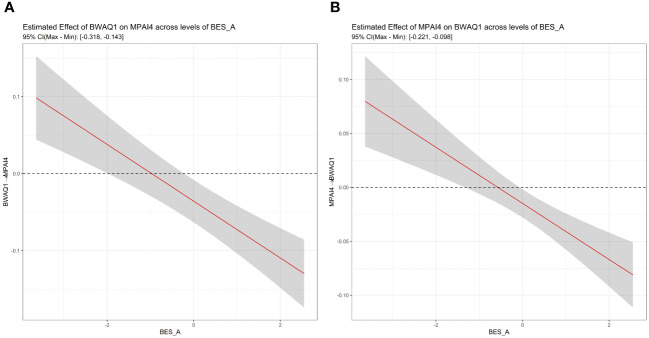
**(A)** The plot of conditional marginal effects of BWAQ1 × BES_A on MPAI4. **(B)** The plot of conditional marginal effects of MPAI4 × BES_A on BWAQ1.

**Figure 3** shows the relationship between PSU and aggression at various affective empathy levels. As expected, “physical aggression” had a positive conditional effect on “productivity loss” when the value of affective empathy is two standard deviations below the mean value, but a negative conditional effect on “productivity loss” when its value is two standard deviations above the mean value (part A of **Figure 3**). Meanwhile, “productivity loss” can, in turn, positively predict “physical aggression” (part B of **Figure 3**) when the value of affective empathy is two standard deviations below the mean value but negatively predict “physical aggression” when its value is two standard deviations above the mean value.

### 3.3 Network stability

The case-dropping subset bootstrap procedure quantifies the stability and centrality estimates of the edge weights, as shown in **Supplementary Figure S4**. All *CS-C*s obtained by the case-dropping test are summarized in **Table 2**. All *CS-C*s exceeded 0.25, indicating that the stability of the results of this study was acceptable.

**Table 2:** Summary of correlation stability coefficients.

Model		Edge weight	*EI*
Affective empathy moderated model	Pairwise	0.75	0.75
	Interactions	0.28	0.44
Cognitive empathy moderated model	Pairwise	0.75	0.75
	Interactions	0.52	0.28

## 4 Discussion

This study examines the bidirectional relationship between PSU and aggressive behavior, taking into account affective and cognitive empathy in secondary school students. It is the first study to use the MNM to measure empathy, PSU, and aggression in a sample of 2,469 secondary school students. As a result, a key set of findings emerged that need to be discussed in detail.

### 4.1 The bidirectional relationship between aggression and PSU

We identified several edges correlating the symptoms of aggression and PSU, namely “hostility” and “withdrawal/escape”, “physical aggression” and “withdrawal/escape”. This finding partially supported hypothesis 1, which suggested that aggression and PSU may interact bidirectionally. Several recent studies also consistently suggested that excessive smartphone use is positively associated with aggression (8), and aggression is strongly associated with excessive mobile phone use (20).

Furthermore, our results also showed that “withdrawal/escape” served as a PSU symptom that correlated with several different aggression symptoms, namely “hostility”, “anger”, and “physical aggression”, which may suggest that “withdrawal/escape” plays an important role in the relationship between PSU and aggression. “Withdrawal/escape” refers to the tendency to use mobile phones to avoid negative feelings (e.g., loneliness) (38), which is related to the inability to be alone. Considering the compensation theory of media use, adolescents who cannot bear to be alone may tend to use mobile phones to temporarily escape feelings of loneliness, which is useless in dealing with negative feelings in the long run (17), and therefore may lead to accumulating anger and hostility and increasing aggressive behavior. A previous study also found that adolescents addicted to mobile phones with poor solitude skills were at higher risk of psychological distress (including depression, anxiety, and stress) (47), which may also contribute to aggression.

More specifically, in this network, the strongest relationship between aggression and PSU was between “withdrawal/escape” and “hostility”. According to the theory of escape motivation (48), individuals commonly engage in passive behaviors such as withdrawal or escape (i.e., a symptom of PSU) to alleviate their discomfort when dealing with stress or anxiety. These behaviors are used to escape their loneliness in the real world (49). However, if they feel that their withdrawal or escape attempts have not alleviated their discomfort, they may become hostile in order to protect themselves or assert dominance (50). Furthermore, an individual’s tendency to constantly withdraw or engage in escape behaviors may be reinforced if they develop a hostile attitude when coping with anxiety, stress, and other negative emotions (16, 51).

### 4.2 The bidirectional relationship between aggression and PSU when moderating empathy

In partial agreement with our hypothesis 2, the result of the current study suggested that affective empathy moderated the relationship between symptoms of aggression and PSU. In contrast, cognitive empathy did not play a moderating role in the relationship between aggression and PSU. Specifically, at higher levels of affective empathy, “productivity loss” and “physical aggression” may interact negatively, whereas at lower levels of affective empathy, the relationship between “productivity loss” and “physical aggression” is positive.

Affective empathy is an emotional or affective component related to the ability to recognize and experience the emotional states of others (25). The inverse relationship between “productive loss” and “physical aggression” at different levels of affective empathy may be related to the different mechanisms of PSU. In early 1992, Finn (52) identified two pathways in media addiction: social compensation and mood management. Previous studies have suggested that affective empathy may serve as a risk factor for depression, meaning that adolescents with higher levels of affective empathy are more vulnerable to negative feelings (e.g., loneliness) and emotional symptoms of depression (53). Given that adolescents may use mobile phones to escape their unpleasant feelings and manage their mood (49), it is understandable that some adolescents with higher levels of affective empathy may perceive more negative feelings and be more likely to suffer from PSU, which may affect their ability to focus on their academic work (54). Meanwhile, affective empathy may serve as a protective factor that reduces physical aggression in adolescents (55), suggesting that adolescents with higher affective empathy may engage in less physically aggressive behavior. Therefore, it is understandable that physical aggression and productive loss may be negatively related in adolescents with higher levels of affective empathy.

Differently, adolescents with lower levels of affective empathy may have difficulties processing emotional information about others and expressing feelings appropriately (56), which may prevent them from developing satisfying interpersonal relationships. Furthermore, lower levels of affective empathy are also associated with more physical aggression (55), which may also contribute to the impairment of interpersonal relationships. Therefore, when social needs are not met, adolescents with lower affective empathy may tend to spend more time on mobile phones to seek compensation (17), which may increase the risk of PSU and threaten their academic productivity. However, problematic mobile phone use may lead to increased negative emotions (e.g., depression and social anxiety) (57), which in turn may increase the likelihood of displaying physical aggression (58). Therefore, the relationship between physical aggression and productivity loss is positive among adolescents with lower affective empathy.

In conclusion, the mechanism of PSU among adolescents with higher affective empathy may be mainly attributed to the mood management route, whereas the mechanism among adolescents with lower affective empathy may be mainly attributed to the social compensation route, which may be related to the different relationships between aggression and PSU at different levels of affective empathy.

## 5 Limitations

The current study has several limitations. First, we found that cognitive empathy had no moderating effect on the relationship between aggression and PSU, which is inconsistent with a large part of previous studies. Therefore, the explanation for the null result in our study could not be more cautious. Moreover, some recent studies tended to divide aggression into two subtypes according to the motivation of aggression: proactive aggression and reactive aggression (30), which is different from the current study. Using motivation to understand behavior may deepen our understanding of aggression, which deserves further investigation.

## 6 Conclusions

In the current study, we use a moderate network approach to analyze the relationship between aggression and PSU, as well as the moderating role of empathy. The results partially supported the bidirectional relationship between PSU and aggression. Meanwhile, we compared the different moderating effects of cognitive empathy and affective empathy. We found that affective empathy could moderate the relationship between two symptoms of aggression and PSU: “productivity loss” and “physical aggression”. Therefore, individuals can employ psychological counseling techniques and skills, such as mindfulness (59), which have been demonstrated to effectively enhance empathy, to address the relationship between PSU and aggression. In sum, the present study suggests that the mechanism of PSU may be different among adolescents with different levels of empathy, and studies to further clarify the underlying mechanism of PSU are still warranted.

## Data availability statement

The raw data supporting the conclusions of this article will be made available by the authors, without undue reservation.

## Ethics statement

This research was reviewed and approved by the Ethics Committee of Beijing Normal University (reference number: 202112220085). The studies were conducted in accordance with the local legislation and institutional requirements. The participants provided their written informed consent to participate in this study.

## Author contributions

WW: Formal analysis, Writing – original draft. XZ: Methodology, Writing – review & editing. QT: Formal analysis, Writing – review & editing. YT: Methodology, Writing – review & editing. SW: Methodology, Writing – review & editing. ZM: Supervision, Writing – review & editing. ML: Conceptualization, Project administration, Supervision, Writing – review & editing. GL: Conceptualization, Supervision, Writing – review & editing.

## References

[1] Sharma G. Mobile phone addiction among children and adolescents. J Addict Nurs. (2019) 30:261–8. doi: 10.1097/JAN.0000000000000309 31800517

[2] Puente M Balmori A. Addiction to cell phones. Are there neurophysiological mechanisms involved? Proyecto. (2007) 61:8–12.

[3] Pavithra MB Madhukumar S Mahadeva Murthy TS. A study on nomophobia - mobile phone dependence, among students of a medical college in Bangalore. Natl J Community Med. (2015) 6:340–4.

[4] Rong FJ Wang MN Peng C Cheng JH Ding HL Wang Y. Association between problematic smartphone use, chronotype and nonsuicidal self-injury among adolescents: A large-scale study in China. Addictive Behav. (2023) 144:107725. doi: 10.1016/j.addbeh.2023.107725 37087768

[5] Yang X Zhou Z Liu Q Fan C. Mobile Phone Addiction and Adolescents’ Anxiety and Depression: The Moderating Role of Mindfulness. J Child Fam Stud. Springer (2019) 28:822–30. doi: 10.1007/s10826-018-01323-2

[6] Samaha M Hawi NS. Relationships among smartphone addiction, stress, academic performance, and satisfaction with life. Comput Hum Behav. (2016) 57:321–5. doi: 10.1016/j.chb.2015.12.045

[7] Jun S. The reciprocal longitudinal relationships between mobile phone addiction and depressive symptoms among Korean adolescents. Comput Hum Behav. (2016) 58:179–86. doi: 10.1016/j.chb.2015.12.061

[8] Karaoglan Yilmaz FG Avci U Yilmaz R. The role of loneliness and aggression on smartphone addiction among university students. Curr Psychol. (Advance online publication, New Brunswick, N.J.) (2022), 1–9. doi: 10.1007/s12144-022-03018-w PMC8933130 35340690

[9] Buss AH Perry M. The aggression questionnaire. J Pers Soc Psychol. (1992) 63:452–9. doi: 10.1037/0022-3514.63.3.452 1403624

[10] Lickley RA Sebastian CL. The neural basis of reactive aggression and its development in adolescence. Psychol Crime Law. (2018) 24:313–33. doi: 10.1080/1068316x.2017.1420187

[11] Peng C Guo TY Cheng JH Wang MN Rong FJ Zhang SY. Sex differences in association between Internet addiction and aggression among adolescents aged 12 to 18 in mainland of China. J Affect Disord. (2022) 312:198–207. doi: 10.1016/j.jad.2022.06.026 35728679

[12] Buchmann A Hohmann S Brandeis D Banaschewski T Poustka L. Aggression in children and adolescents. In: Miczek KA Meyer-Lindenberg A, (Eds.), Neuroscience of Aggression. Springer-Verlag Publishing (2014). p. 421–42. doi: 10.1007/7854_2013_261 24362971

[13] José D Fernando R Gabriel R. Cell-phone addiction: A review. Front Psychiatry. (2016) 7:175. doi: 10.3389/fpsyt.2016.00175 27822187 PMC5076301

[14] Reppucci C Petrovich G. Organization of connections between the amygdala, medial prefrontal cortex, and lateral hypothalamus: a single and double retrograde tracing study in rats. Brain Structure Funct. (2016) 221:2937–62. doi: 10.1007/s00429-015-1081-0 PMC4713378 26169110

[15] Denson TF DeWall CN Finkel EJ. Self-control and aggression. Curr Dir psychol Sci. (2012) 21:20–5. doi: 10.1177/0963721411429451

[16] Roberton T Daffern M Bucks RS. Emotion regulation and aggression. Aggression Violent Behav. (2012) 17:72–82. doi: 10.1016/j.avb.2011.09.006

[17] Park WK. Mobile phone addiction. In: Mobile communications: Re-negotiation of the social sphere. Springer London, London (2005). p. 253–72.

[18] Jiang ZC Zhao XX. Self-control and problematic mobile phone use in Chinese college students: the mediating role of mobile phone use patterns. BMC Psychiatry. (2016) 16:416–6. doi: 10.1186/s12888-016-1131-z PMC5120559 27876032

[19] Miller NE Sears RR Mowrer HO Doob LW Dollard J. The frustration-aggression hypothesis. psychol Rev. (1941) 48:337–42. doi: 10.1037/h0055861

[20] Sahu M Gandhi S Sharma MK. Mobile phone addiction among children and adolescents: A systematic review. J Addict Nurs. (2019) 30:261–8. doi: 10.1097/JAN.0000000000000309 31800517

[21] Lakens D. Using a smartphone to measure heart rate changes during relived happiness and anger. IEEE Trans Affect Computing. (2013) 4:238–41. doi: 10.1109/T-AFFC.2013.3

[22] Chester DSJS Compass PP. Aggression as successful self-control. Soc Pers Psychol Compass. Advance online publication (2023):e12832. doi: 10.1111/spc3.12832

[23] Vachon DD Lynam DR Johnson JA. The (Non)Relation between empathy and aggression: surprising results from a meta-analysis. psychol Bull. (2014) 140:751–73. doi: 10.1037/a0035236 24364745

[24] Hao Z Jin L Lyu R Rabia Akram H. Problematic mobile phone use and altruism in Chinese undergraduate students: The mediation effects of alexithymia and empathy. Children Youth Serv Rev. (2020) 118:105402. doi: 10.1016/j.childyouth.2020.105402

[25] Spinrad TL Eisenberg N Morris AS. Empathy-related responding in children. In Killen N Smetana JG. (Eds.) Handbook of moral development. Routledge (2023). p. 255–71.

[26] Swit CS Harty SC. Normative beliefs and aggression: the mediating roles of empathy and anger. Child Psychiatry Hum Dev. Advance online publication (2023), 1–13. doi: 10.1007/s10578-023-01558-1 PMC11828841 37347363

[27] Mak B Nickerson RC Sim J. Mobile technology dependence and mobile technostress. Int J Innovation Technol Manage. (2018) 15:1850039. doi: 10.1142/S0219877018500396

[28] Vachon DD Lynam DR. Fixing the problem with empathy: development and validation of the affective and cognitive measure of empathy. Assessment. (2015) 23:135–49. doi: 10.1177/1073191114567941 25612628

[29] van Langen MAM Wissink IB van Vugt ES van der Stouwe T Stams GJJM. The relation between empathy and offending: A meta-analysis. Aggression Violent Behav. (2014) 19:179–89. doi: 10.1016/j.avb.2014.02.003

[30] Chen FR Fung ALC Raine A. The cognitive, affective, and somatic empathy scales (CASES): Cross-cultural replication and specificity to different forms of aggression and victimization. J Pers Assess. (2021) 103:80–91. doi: 10.1080/00223891.2019.1677246 31633394

[31] Wu J Yang Z Wu M Huang H. The relationship between college students’ mobile phone addiction and aggression: A moderated mediation model. Appl Res Qual Life. (2022) 18:1037–55. doi: 10.1007/s11482-022-10126-z

[32] Borsboom D Cramer AO. Network analysis: an integrative approach to the structure of psychopathology. Annu Rev Clin Psychol. (2013) 9:91–121. doi: 10.1146/annurev-clinpsy-050212-185608 23537483

[33] Tao Y Niu H Li Y Liu X Wang S Ma Z. Effects of personal relative deprivation on the relationship between anger rumination and aggression during and after the COVID-19 pandemic lockdown: A longitudinal moderated network approach. J Adolescence. (2023) 95:596–608. doi: 10.1002/jad.12140 36638841

[34] Jolliffe D Farrington DP. Development and validation of the basic empathy scale. J Adolescence. (2006) 29:589–611. doi: 10.1016/j.adolescence.2005.08.010 16198409

[35] Geng Y Xia D Qin BJCP Development H. The Basic Empathy Scale: A Chinese validation of a measure of empathy in adolescents. Child Psychiatry Hum Dev. (2012) 43:499–510. doi: 10.1007/s10578-011-0278-6 22222487

[36] Buss AH Warren W. Aggression questionnaire:(AQ). Torrance, CA: Western Psychological Services (2000).

[37] Maxwell JPJP Differences I. Psychometric properties of a Chinese version of the Buss–Warren Aggression Questionnaire. Pers Individ Dif. (2008) 44:943–53. doi: 10.1016/j.paid.2007.10.037

[38] Leung L. Linking psychological attributes to addiction and improper use of the mobile phone among adolescents in Hong Kong. J Children Media. (2008) 2:93–113. doi: 10.1080/17482790802078565

[39] Li XW Feng XC Xiao WL Zhou H. Loneliness and mobile phone addiction among Chinese college students: the mediating roles of boredom proneness and self-control. Psychol Res Behav Manage. (2021) 14:687–94. doi: 10.2147/prbm.S315879 PMC8206064 34149289

[40] R Core Team. A Language and Environment for Statistical Computing. (2022). Available at: https://www.R-project.org/.

[41] Friedman J Hastie T Tibshirani R. Sparse inverse covariance estimation with the graphical lasso. Biostatistics (Oxford England). (2008) 9:432–41. doi: 10.1093/biostatistics/kxm045 PMC3019769 18079126

[42] Ravikumar P Wainwright MJ Lafferty JD. High-dimensional Ising model selection using ℓ 1-regularized logistic regression. Ann Stat. (2010) 38:1287–319. doi: 10.1214/09-AOS691

[43] Haslbeck J Waldorp LJ. mgm: Estimating time-varying mixed graphical models in high-dimensional data. J Stat Softw. (2015) 93:1–46. doi: 10.18637/jss.v093.i08

[44] Epskamp S Waldorp LJ Mõttus R Borsboom D. The Gaussian graphical model in cross-sectional and time-series data. Multivariate Behav Research. (2018) 53:453–80. doi: 10.1080/00273171.2018.1454823 29658809

[45] Robinaugh DJ Millner AJ McNally RJ. Identifying highly influential nodes in the complicated grief network. J Abnormal Psychol. (2016) 125:747–57. doi: 10.1037/abn0000181 PMC5060093 27505622

[46] Epskamp S Borsboom D Fried EI. Estimating psychological networks and their accuracy: A tutorial paper. Behav Res Methods. (2018) 50:195–212. doi: 10.3758/s13428-017-0862-1 28342071 PMC5809547

[47] Lian SL Sun XJ Niu GF Yang XJ Zhou ZK Yang C. Mobile phone addiction and psychological distress among Chinese adolescents: The mediating role of rumination and moderating role of the capacity to be alone. J Affect Disord. (2021) 279:701–10. doi: 10.1016/j.jad.2020.10.005 PMC7539895 33197839

[48] Kim J-H. Smartphone-mediated communication vs. face-to-face interaction: Two routes to social support and problematic use of smartphone. Comput Hum Behav. (2017) 67:282–91. doi: 10.1016/j.chb.2016.11.004

[49] Li JY Zhan DN Zhou YH Gao XM. Loneliness and problematic mobile phone use among adolescents during the COVID-19 pandemic: The roles of escape motivation and self-control. Addictive Behav. (2021) 118:106857. doi: 10.1016/j.addbeh.2021.106857 PMC8598166 33676160

[50] Lindsay JJ Anderson CA. From antecedent conditions to violent actions: A general affective aggression model. Pers Soc Psychol Bull. (2000) 26:533–47. doi: 10.1177/0146167200267002

[51] Hofmann SG Sawyer AT Fang A Asnaani A. Emotion dysregulation model of mood and anxiety disorders. Depression Anxiety. (2012) 29:409–16. doi: 10.1002/da.21888 22430982

[52] Finn S. Television “Addiction?” An evaluation of four competing media-use models. Journalism Q. (1992) 69:422–35. doi: 10.1177/107769909206900216

[53] Yan ZQ Zeng X Su JL Zhang XX. The dark side of empathy: Meta-analysis evidence of the relationship between empathy and depression. Psych J. (2021) 10:794–804. doi: 10.1002/pchj.482 34494388

[54] Yadav MS Kodi SM Deol R. Impact of mobile phone dependence on behavior and academic performance of adolescents in selected schools of Uttarakhand, India. J Educ Health Promotion. (2021) 10:327–7. doi: 10.4103/jehp.jehp_915_20 PMC8552249 34761013

[55] Yeo LS Ang RP Loh S Fu KJ Karre JK. The role of affective and cognitive empathy in physical, verbal, and indirect aggression of a Singaporean sample of boys. J Psychol. (2011) 145:313–30. doi: 10.1080/00223980.2011.568986 21834324

[56] Decety J Cowell JM. Empathy, Justice, and Moral Behavior. AJOB Neuroscience. (2015) 6(3):3–14. doi: 10.1080/21507740.2015.1047055 PMC4748844 26877887

[57] Chen L Yan Z Tang WJ Yang FY Xie XD He JC. Mobile phone addiction levels and negative emotions among Chinese young adults: The mediating role of interpersonal problems. Comput Hum Behav. (2016) 55:856–66. doi: 10.1016/j.chb.2015.10.030

[58] Andrews LA Brothers SL Sauve JS Nangle DW Erdley CA Hord MK. Fight and flight: Examining putative links between social anxiety and youth aggression. Aggression Violent Behav. (2019) 48:94–103. doi: 10.1016/j.avb.2019.08.005

[59] Hu Z Wen Y Wang Y Lin Y Shi J Yu Z. Effectiveness of mindfulness-based interventions on empathy: A meta-analysis. Front Psychol. (2022) 13:992575. doi: 10.3389/fpsyg.2022.992575 36337535 PMC9632989

