# Supplementary material for: Effects of empathy on the bidirectional relationships between problematic smartphone use and aggression among secondary school students: a moderated network approach
Source: Front Psychiatry. 2024 Feb 29;15:1359932. doi: 10.3389/fpsyt.2024.1359932 (PMC10962280; doi:10.3389/fpsyt.2024.1359932)
Supplement: Supplementary file 1 [file DataSheet_1.docx]

**Statistical steps:**

First, in order to construct a relatively sparse network structure to control spurious relationships and reduce the rate of false-positive associations, the model selection procedure was implemented to determine which variables should be included in the final model. The hierarchical LASSO method in the R package *modnets* (Swanson, 2020) can integrate hierarchical constraints into the LASSO and compute regularization path along a grid of values for the regularization parameter lambda, which affords automated variable and interaction selection in moderated networks (Bien et al., 2013; Lim & Hastie, 2015). Bayesian information criterion (BIC) was used to select the best regularization parameter lambda.

Second, we used the function *fitNetwork* in the *modnets* package to estimate the network structure based on the variable selection object. It should be noted that when moderators are included in the model, some traditional methods of parameter estimation in regression analysis, such as the maximum likelihood estimation, are no longer applicable (Swanson, 2020). Therefore, the *fitNetwork* function used the approach called nodewise regression (Epskamp, Waldorp, et al., 2018; Haslbeck & Waldorp, 2020), which entails estimating the network structure via a series of univariate regression models (Meinshausen & Bühlmann, 2006).

*PlotNet* function was applied to visualize the network. There are two rules in the *plotNet* function: AND rule and OR rule. With the AND rule, we only draw an edge if the regression coefficients of two variables predicting each other are both significant. With the OR rule, the edge will be presented whenever one of the two variables predicting each other is significant. Green lines indicate a positive relationship between two variables, red lines indicate negative relationships. Dashed lines indicate that the relationship between two variables is moderated by the moderating variable significantly, while the solid line means that the moderating effect is not significant. In the current study, all network structures were visualized using the AND rule.

Finally, the case-dropping bootstrap was used to quantify the stability of edge weights and centrality estimates (Epskamp, Borsboom, et al., 2018). The correlation between the original and smaller subset sample, with up to 75% of individuals removed, is evaluated in this approach. Correlation stability coefficients (*CS-C*) were developed to quantify the parameters’ stability. The *CS-C* represents the greatest number of cases that may be dropped with 95% certainty while maintaining a correlation of 0.70 or higher with the original parameters. It is reasonable to have a *CS*-*C* at least .25, preferably above .50 (Costenbader & Valente, 2003).

**References**

Bien, J., Taylor, J., & Tibshirani, R. (2013). A LASSO FOR HIERARCHICAL INTERACTIONS. *Annals of Statistics*, *41*(3), 1111–1141. https://doi.org/10.1214/13-AOS1096

Costenbader, E., & Valente, T. W. (2003). The stability of centrality measures when networks are sampled. *Social Networks*, *25*(4), 283–307. https://doi.org/10.1016/S0378-8733(03)00012-1

Epskamp, S., Borsboom, D., & Fried, E. I. (2018). Estimating psychological networks and their accuracy: A tutorial paper. *Behavior Research Methods*, *50*(1), 195–212. https://doi.org/10.3758/s13428-017-0862-1

Epskamp, S., Waldorp, L. J., Mõttus, R., & Borsboom, D. (2018). The Gaussian Graphical Model in Cross-Sectional and Time-Series Data. *Multivariate Behavioral Research*, *53*(4), 453–480. https://doi.org/10.1080/00273171.2018.1454823

Haslbeck, J. M. B., & Waldorp, L. J. (2020). mgm: Estimating Time-Varying Mixed Graphical Models in High-Dimensional Data. *Journal of Statistical Software*, *93*, 1–46. https://doi.org/10.18637/jss.v093.i08

Lim, M., & Hastie, T. (2015). Learning Interactions via Hierarchical Group-Lasso Regularization. *Journal of Computational and Graphical Statistics*, *24*(3), 627–654. https://doi.org/10.1080/10618600.2014.938812

Meinshausen, N., & Bühlmann, P. (2006). High-dimensional graphs and variable selection with the Lasso. *The Annals of Statistics*, *34*(3), 1436–1462. https://doi.org/10.1214/009053606000000281

Swanson, T. J. (2020). *Modeling Moderators in Psychological Networks* [Doctor, University of Kansas]. https://www.proquest.com/openview/d151ab6b93ad47e3f0d5e59d7b6fd3d3/1?pq-origsite=gscholar&cbl=44156

**Table S1.** Variable selection results through *varSelect* function under the condition that the moderator was affective empathy.

|  | **MPAI1** | **MPAI2** | **MPAI3** | **MPAI4** | **BWAQ1** | **BWAQ2** | **BWAQ3** | **BWAQ4** | **BWAQ5** |
| --- | --- | --- | --- | --- | --- | --- | --- | --- | --- |
| **mods** | MPAI2 | MPAI1 | MPAI1 | MPAI1 | MPAI2 | MPAI3 | BWAQ1 | MPAI1 | MPAI3 |
|  | MPAI3 | MPAI3 | MPAI2 | MPAI2 | MPAI3 | BWAQ1 | BWAQ2 | MPAI4 | BWAQ1 |
|  | MPAI4 | MPAI4 | MPAI4 | MPAI3 | MPAI4 | BWAQ3 | BWAQ4 | BWAQ1 | BWAQ2 |
|  | BWAQ3 | BWAQ1 | BWAQ1 | BWAQ1 | BWAQ2 | BWAQ4 | BWAQ5 | BWAQ2 | BWAQ3 |
|  | BWAQ4 | BES_A | BWAQ2 | BWAQ4 | BWAQ3 | BWAQ5 | BES_A | BWAQ3 | BWAQ4 |
|  | BES_A |  | BWAQ3 | BES_A | BWAQ4 | BES_A |  | BWAQ5 | BES_A |
|  |  |  | BWAQ5 |  | BWAQ5 |  |  | BES_A |  |
|  |  |  | BES_A |  | BES_A |  |  |  |  |
| **ints** | MPAI3:BES_A | BWAQ1:BES_A | MPAI4:BES_A | BWAQ1:BES_A | MPAI2:BES_A | BWAQ5:BES_A |  | BWAQ1:BES_A | BWAQ2:BES_A |
|  | MPAI4:BES_A |  | BWAQ1:BES_A |  | MPAI4:BES_A |  |  | BWAQ2:BES_A | BWAQ3:BES_A |
|  | BWAQ3:BES_A |  | BWAQ3:BES_A |  | BWAQ2:BES_A |  |  | BWAQ3:BES_A |  |
|  |  |  |  |  | BWAQ4:BES_A |  |  |  |  |
|  |  |  |  |  | BWAQ5:BES_A |  |  |  |  |

**Table S2**. Variable selection results through *varSelect* function under the condition that the moderator was cognitive empathy.

|  | **MPAI1** | **MPAI2** | **MPAI3** | **MPAI4** | **BWAQ1** | **BWAQ2** | **BWAQ3** | **BWAQ4** | **BWAQ5** |
| --- | --- | --- | --- | --- | --- | --- | --- | --- | --- |
| **mods** | MPAI2 | MPAI1 | MPAI1 | MPAI1 | MPAI2 | MPAI3 | MPAI1 | MPAI1 | MPAI3 |
|  | MPAI3 | MPAI3 | MPAI2 | MPAI2 | MPAI3 | BWAQ1 | MPAI3 | MPAI4 | BWAQ1 |
|  | MPAI4 | MPAI4 | MPAI4 | MPAI3 | BWAQ2 | BWAQ3 | BWAQ1 | BWAQ1 | BWAQ2 |
|  | BWAQ3 | BWAQ1 | BWAQ1 | BWAQ4 | BWAQ3 | BWAQ4 | BWAQ2 | BWAQ2 | BWAQ3 |
|  | BWAQ4 | BES_C | BWAQ2 | BES_C | BWAQ4 | BWAQ5 | BWAQ4 | BWAQ3 | BWAQ4 |
|  | BES_C |  | BWAQ4 |  | BWAQ5 | BES_C | BWAQ5 | BWAQ5 | BES_C |
|  |  |  | BWAQ5 |  | BES_C |  | BES_C | BES_C |  |
|  |  |  | BES_C |  |  |  |  |  |  |
| **ints** | MPAI2:BES_C | MPAI1:BES_C | MPAI4:BES_C | MPAI1:BES_C | BWAQ3:BES_C | BWAQ3:BES_C | BWAQ1:BES_C | MPAI4:BES_C | MPAI3:BES_C |
|  | MPAI4:BES_C | MPAI3:BES_C | BWAQ1:BES_C | MPAI3:BES_C | BWAQ4:BES_C |  | BWAQ2:BES_C | BWAQ1:BES_C |  |
|  |  |  | BWAQ2:BES_C |  |  |  | BWAQ5:BES_C | BWAQ2:BES_C |  |
|  |  |  | BWAQ4:BES_C |  |  |  |  | BWAQ5:BES_C |  |
|  |  |  | BWAQ5:BES_C |  |  |  |  |  |  |

**Table S3.** The adjacency matrix of affective empathy moderated network (*p* < .05).

|  | **MPAI1** | **MPAI2** | **MPAI3** | **MPAI4** | **BWAQ1** | **BWAQ2** | **BWAQ3** | **BWAQ4** | **BWAQ5** |
| --- | --- | --- | --- | --- | --- | --- | --- | --- | --- |
| **MPAI1** | 0.000 | 0.349 | 0.072 | 0.535 | 0.000 | 0.000 | 0.000 | 0.037 | 0.000 |
| **MPAI2** | 0.349 | 0.000 | 0.413 | 0.168 | 0.069 | 0.000 | 0.000 | 0.000 | 0.000 |
| **MPAI3** | 0.072 | 0.413 | 0.000 | 0.183 | -0.079 | 0.050 | 0.000 | 0.000 | 0.152 |
| **MPAI4** | 0.535 | 0.168 | 0.183 | 0.000 | -0.025 | 0.000 | 0.000 | 0.050 | 0.000 |
| **BWAQ1** | 0.000 | 0.069 | -0.079 | -0.025 | 0.000 | 0.214 | 0.041 | 0.508 | 0.089 |
| **BWAQ2** | 0.000 | 0.000 | 0.050 | 0.000 | 0.214 | 0.000 | 0.320 | 0.170 | 0.449 |
| **BWAQ3** | 0.000 | 0.000 | 0.000 | 0.000 | 0.041 | 0.320 | 0.000 | 0.063 | 0.251 |
| **BWAQ4** | 0.037 | 0.000 | 0.000 | 0.050 | 0.508 | 0.170 | 0.063 | 0.000 | 0.199 |
| **BWAQ5** | 0.000 | 0.000 | 0.152 | 0.000 | 0.089 | 0.449 | 0.251 | 0.199 | 0.000 |

**Table S4.** The matrix of interaction terms associated with affective empathy moderated network (*p* < .05).

|  | **MPAI1** | **MPAI2** | **MPAI3** | **MPAI4** | **BWAQ1** | **BWAQ2** | **BWAQ3** | **BWAQ4** | **BWAQ5** |
| --- | --- | --- | --- | --- | --- | --- | --- | --- | --- |
| **MPAI1 : BES_A** | 0.000 | 0.000 | 0.000 | 0.000 | 0.000 | 0.000 | 0.000 | 0.000 | 0.000 |
| **MPAI2 : BES_A** | 0.000 | 0.000 | 0.000 | 0.000 | 0.000 | 0.000 | 0.000 | 0.000 | 0.000 |
| **MPAI3 : BES_A** | 0.000 | 0.000 | 0.000 | 0.000 | 0.000 | 0.000 | 0.000 | 0.000 | 0.000 |
| **MPAI4 : BES_A** | 0.000 | 0.000 | 0.000 | 0.000 | -0.026 | 0.000 | 0.000 | 0.000 | 0.000 |
| **BWAQ1 : BES_A** | 0.000 | 0.000 | 0.000 | -0.037 | 0.000 | 0.000 | 0.000 | 0.000 | 0.000 |
| **BWAQ2 : BES_A** | 0.000 | 0.000 | 0.000 | 0.000 | 0.000 | 0.000 | 0.000 | 0.000 | 0.000 |
| **BWAQ3 : BES_A** | 0.000 | 0.000 | 0.000 | 0.000 | 0.000 | 0.000 | 0.000 | 0.000 | 0.000 |
| **BWAQ4 : BES_A** | 0.000 | 0.000 | 0.000 | 0.000 | 0.000 | 0.000 | 0.000 | 0.000 | 0.000 |
| **BWAQ5 : BES_A** | 0.000 | 0.000 | 0.000 | 0.000 | 0.000 | 0.000 | 0.000 | 0.000 | 0.000 |

**Table S5.** The adjacency matrix of cognitive empathy moderated network.

|  | **MPAI1** | **MPAI2** | **MPAI3** | **MPAI4** | **BWAQ1** | **BWAQ2** | **BWAQ3** | **BWAQ4** | **BWAQ5** |
| --- | --- | --- | --- | --- | --- | --- | --- | --- | --- |
| **MPAI1** | 0.000 | 0.345 | 0.072 | 0.528 | 0.000 | 0.000 | 0.035 | 0.035 | 0.000 |
| **MPAI2** | 0.345 | 0.000 | 0.414 | 0.165 | 0.061 | 0.000 | 0.000 | 0.000 | 0.000 |
| **MPAI3** | 0.072 | 0.414 | 0.000 | 0.194 | -0.070 | 0.044 | 0.000 | 0.000 | 0.145 |
| **MPAI4** | 0.528 | 0.165 | 0.194 | 0.000 | 0.000 | 0.000 | 0.000 | 0.038 | 0.000 |
| **BWAQ1** | 0.000 | 0.061 | -0.070 | 0.000 | 0.000 | 0.206 | 0.069 | 0.504 | 0.089 |
| **BWAQ2** | 0.000 | 0.000 | 0.044 | 0.000 | 0.206 | 0.000 | 0.307 | 0.167 | 0.454 |
| **BWAQ3** | 0.035 | 0.000 | 0.000 | 0.000 | 0.069 | 0.307 | 0.000 | 0.060 | 0.243 |
| **BWAQ4** | 0.035 | 0.000 | 0.000 | 0.038 | 0.504 | 0.167 | 0.060 | 0.000 | 0.205 |
| **BWAQ5** | 0.000 | 0.000 | 0.145 | 0.000 | 0.089 | 0.454 | 0.243 | 0.205 | 0.000 |

**Table S6.** The matrix of interaction terms associated with cognitive empathy moderated network.

|  | **MPAI1** | **MPAI2** | **MPAI3** | **MPAI4** | **BWAQ1** | **BWAQ2** | **BWAQ3** | **BWAQ4** | **BWAQ5** |
| --- | --- | --- | --- | --- | --- | --- | --- | --- | --- |
| **MPAI1 : BES_C** | 0.000 | -0.029 | 0.000 | 0.040 | 0.000 | 0.000 | 0.000 | 0.000 | 0.000 |
| **MPAI2 : BES_C** | -0.044 | 0.000 | 0.000 | 0.000 | 0.000 | 0.000 | 0.000 | 0.000 | 0.000 |
| **MPAI3 : BES_C** | 0.000 | 0.000 | 0.000 | -0.052 | 0.000 | 0.000 | 0.000 | 0.000 | 0.000 |
| **MPAI4 :BES_C** | -0.017 | 0.000 | -0.047 | 0.000 | 0.000 | 0.000 | 0.000 | 0.000 | 0.000 |
| **BWAQ1 : BES_C** | 0.000 | 0.000 | 0.000 | 0.000 | 0.000 | 0.000 | 0.090 | -0.021 | 0.000 |
| **BWAQ2 : BES_C** | 0.000 | 0.000 | 0.000 | 0.000 | 0.000 | 0.000 | -0.068 | 0.000 | 0.000 |
| **BWAQ3 : BES_C** | 0.000 | 0.000 | 0.000 | 0.000 | 0.033 | -0.013 | 0.000 | 0.000 | 0.000 |
| **BWAQ4 : BES_C** | 0.000 | 0.000 | 0.000 | 0.000 | -0.071 | 0.000 | 0.000 | 0.000 | 0.000 |
| **BWAQ5 : BES_C** | 0.000 | 0.000 | 0.000 | 0.000 | 0.000 | 0.000 | 0.000 | 0.000 | 0.000 |


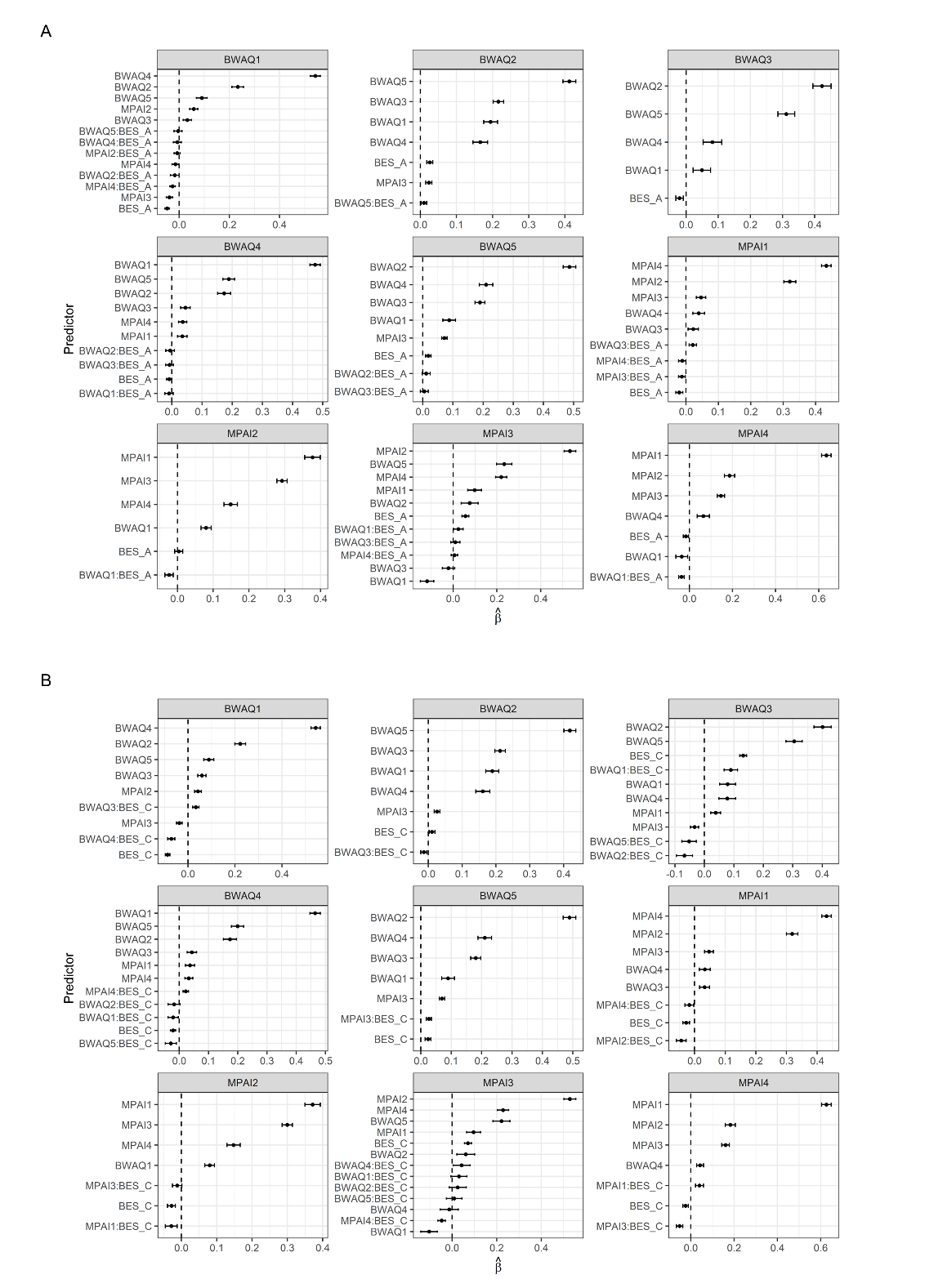
**Figure S1.** Plot or dataframe showing the point estimates from each model, along with confidence intervals based on the estimated standard errors. A: Affective empathy moderated network. B: Cognitive empathy moderated network.

**
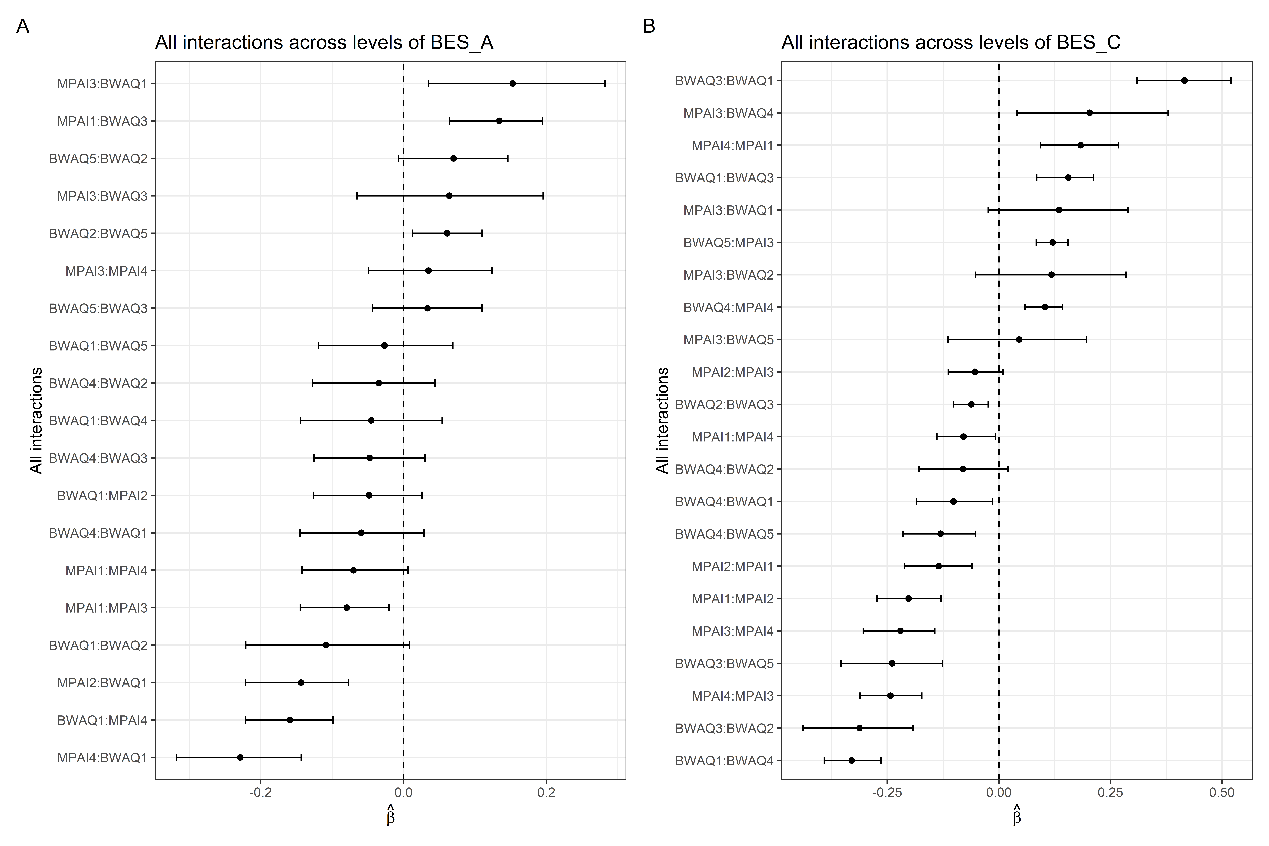
Figure S2.** A: The 95% confidence intervals of all interactions when the moderating variable was affective empathy. B: The 95% confidence intervals of all interactions when the moderating variable was cognitive empathy.


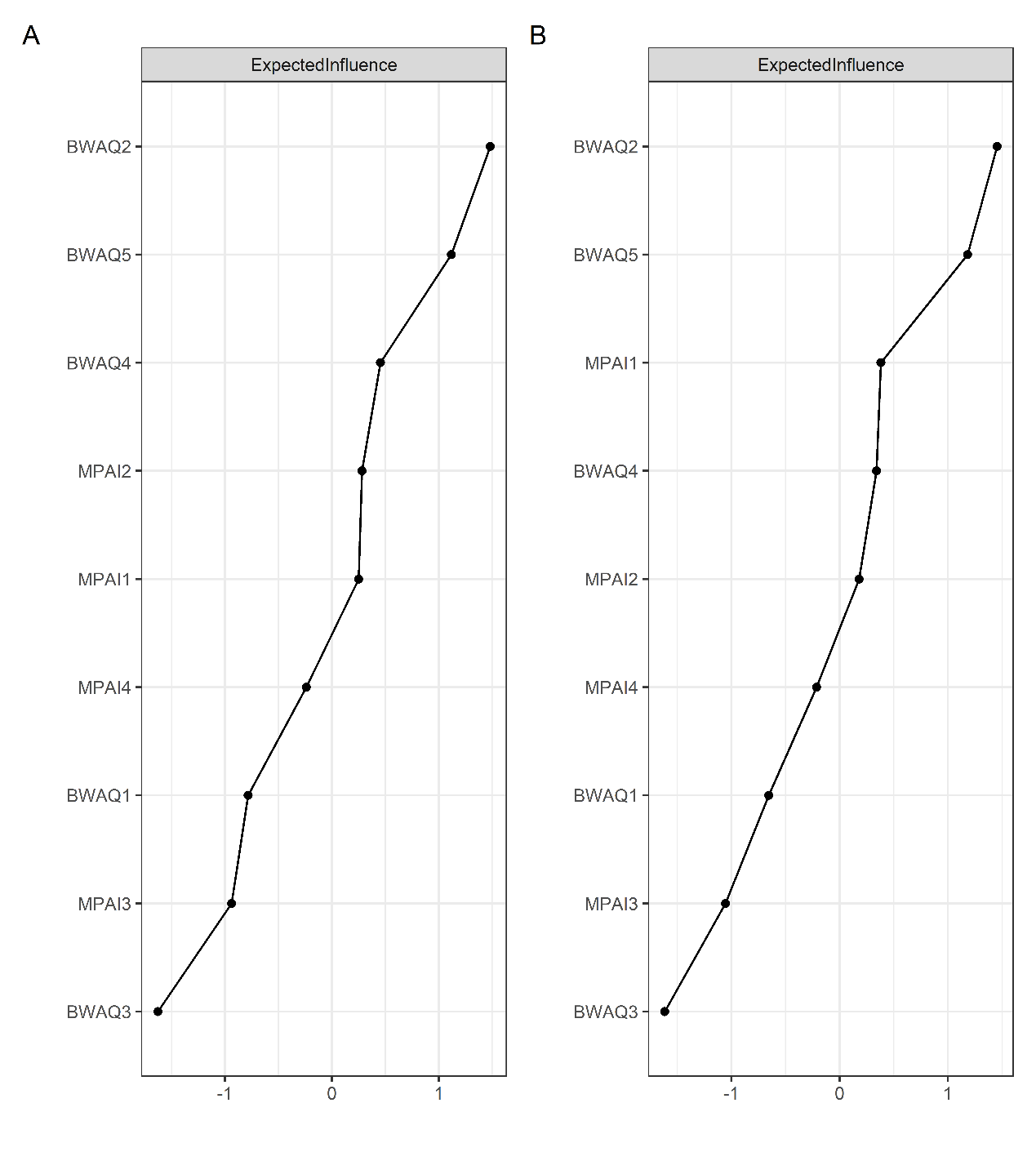
**Figure S3.** The *EI* values (standardized *Z* scores) of all MPAI and BWAQ items in the affective empathy moderated network model (left panel) and the cognitive empathy moderated network model (right panel).

**
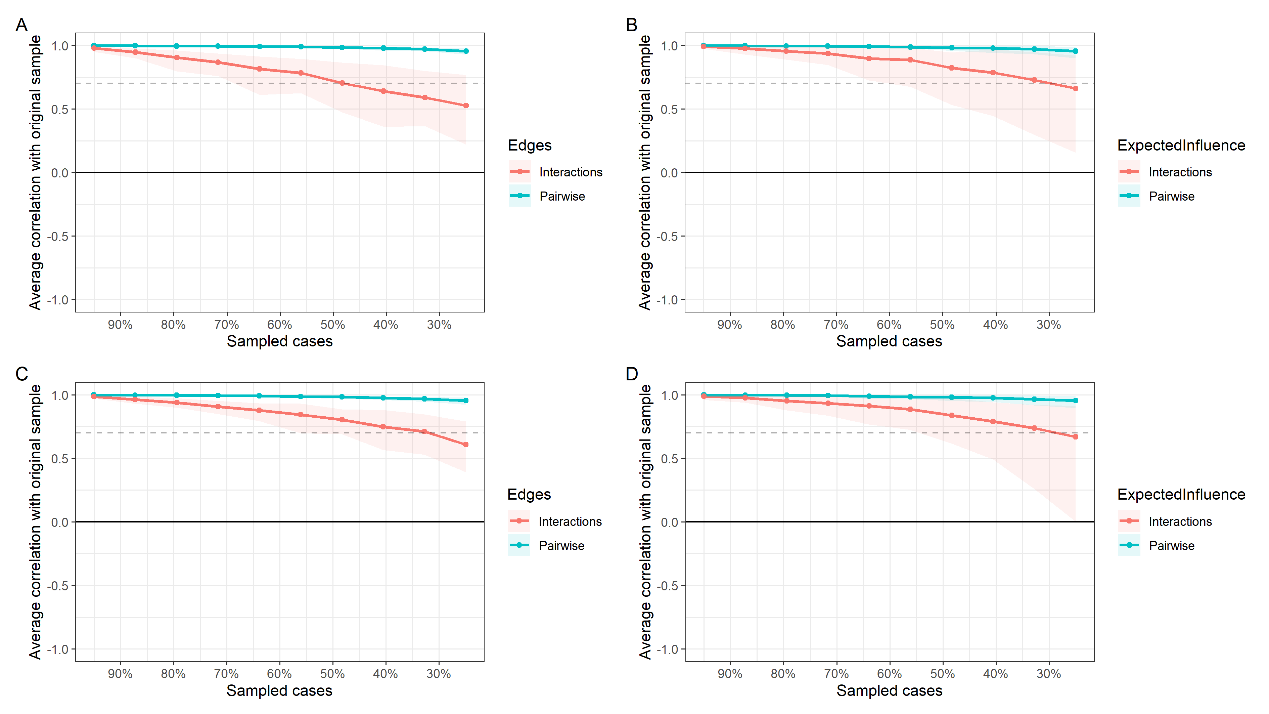
Figure S4.** Case-dropping results (1000 interactions) of two networks. Average correlations were plotted with 95% coverage intervals for each subsample size. A: Edge weights case-dropping results of affective empathy moderated network. B: *EI* case-dropping results of affective empathy moderated network. C: Edge weights case-dropping results of cognitive empathy moderated network. D: *EI* case-dropping results of cognitive empathy moderated network.
